# Involvement of a C-terminal motif in the interference of primate lentiviral Vpu proteins with CD1d-mediated antigen presentation

**DOI:** 10.1038/srep09675

**Published:** 2015-04-15

**Authors:** Susanna M. Bächle, Daniel Sauter, Sabrina Sibitz, Johan K. Sandberg, Frank Kirchhoff, Markus Moll

**Affiliations:** 1 Center for Infectious Medicine, Department of Medicine, Karolinska Institutet, Karolinska University Hospital Huddinge, 14186 Stockholm, Sweden; 2 Institute of Molecular Virology, Ulm University Medical Center, 89081 Ulm, Germany

## Abstract

The HIV-1 accessory protein Vpu is emerging as a critical factor for viral evasion from innate immunity. We have previously shown that the Vpu proteins of two HIV-1 group M subtype B strains (NL4-3 and BaL) down-regulate CD1d from the surface of infected dendritic cells (DCs) and inhibit their crosstalk with the innate invariant natural killer T (iNKT) cells. In the present study, we have investigated the ability of a comprehensive set of primate lentiviral Vpu proteins to interfere with CD1d-mediated immunity. We found that CD1d down-regulation is a conserved function of Vpu proteins from HIV-1 groups M, O and P as well as their direct precursors SIVcpz*Ptt* and SIVgor. At the group M subtype level, subtype C Vpu proteins were significantly weaker CD1d antagonists than subtype B Vpu proteins. Functional characterization of different mutants and chimeras derived from active subtype B and inactive subtype C Vpu proteins revealed that residues in the cytoplasmic domain are important for CD1d down-regulation. Specifically, we identified a C-terminal APW motif characteristic for group M subtype B Vpu proteins necessary for interference with CD1d surface expression. These findings support the notion that Vpu plays an important role in lentiviral evasion from innate immunity.

Innate cell-mediated immune mechanisms play an important role in the host defence against viral infections. Innate cell types, such as natural killer (NK) cells and invariant natural killer T (iNKT) cells, respond to invading pathogens without priming and may thus contribute to controlling viral replication during the earliest stages of infection (reviewed in Refs.^ 1^,^2^). The importance of these cells in the antiviral host defence is underscored by the fact that many viruses, including primate lentiviruses, have evolved mechanisms to evade NK and iNKT cell responses. Human immunodeficiency virus 1 (HIV-1) targets innate immune cells directly but also indirectly by inhibiting ligands activating these cells, and the HIV-1 accessory viral protein U (Vpu) is emerging as a viral factor critically involved in the latter processes (reviewed in Ref. 3). In addition to degradation of the viral receptor CD4 and enhancement of viral particle release via tetherin antagonism^4^,^5^,^6^, Vpu down-regulates the host cell molecules NTB-A (NK-, T-, and B-cell antigen, CD352) and PVR (poliovirus receptor, CD155) to inhibit activation of NK cells^7^,^8^. Moreover, Vpu cooperates with the accessory protein Nef to inhibit activation of iNKT cells by interfering with cell surface expression of CD1d^9^,^10^. CD1d presents lipid antigens of endogenous and exogenous origin to iNKT cells^11^, and is expressed by potential HIV target cells such as dendritic cells and macrophages^12^. So far no virally derived antigens able to activate iNKT cells have been described. In an inflammatory milieu, however, iNKT cells can be activated in the absence of exogenous antigen through the recognition of endogenous CD1d-presented glucopyranosylceramide (GlcCer), which accumulates in response to infection as well as Toll-like receptor stimulation^13^. Moreover, hepatitis B virus (HBV) induces the production of antigenic lipids in the ER of infected hepatocytes^14^. These lipids are presented at the cell surface, presumably in the context of CD1d, enabling detection of HBV-infected cells by NKT cells. Disturbance of lipid metabolism and membrane composition occurs also for other viruses, including HIV-1^15^. Overall, there is evidence for detection of virally infected cells via CD1d, and for an important role for CD1d-restricted iNKT cells in the antiviral defence.

Vpu is an integral membrane protein with a typical length of 77–86 amino acids that folds into distinct structural domains with different biological functions^16^. Early studies showed that the transmembrane domain (TMD) of Vpu is critical for enhancement of virus release from infected cells, while the cytoplasmic domain is important for CD4 degradation^17^. Interference with the expression of CD4 and tetherin requires phosphorylation of Vpu at the serine residues in the highly conserved DSGxxS motif in its cytoplasmic domain^5^,^18^. Whereas mutation of these phosphorylation sites impairs CD4 and tetherin down-regulation from the cell surface, it has no or limited effect on down-regulation of CD1d, NTB-A and PVR^7^,^8^,^19^, suggesting that different functions of Vpu are genetically separable and follow distinct mechanistic pathways. We recently found that Vpu interferes with CD1d surface expression by decreasing the recycling rate of CD1d from endosomal compartments to the cell surface and retaining CD1d in early endosomes^10^. However, our knowledge about the mechanisms of Vpu interference with CD1d is still incomplete, and molecular and structural details remain to be resolved.

The *vpu* open-reading frame is found in both pandemic (M) and non-pandemic (N, O and P) HIV-1 groups, as well as in some closely related simian immunodeficiency virus (SIV) strains (reviewed in Ref. 20). Notably, HIV-1 and SIV Vpu proteins are genetically and functionally diverse, and this may affect the ability of the respective viruses to cross species barriers and to spread in new hosts. Specifically, HIV-1 group M strains that account for the AIDS pandemic encode Vpu proteins that counteract both tetherin and CD4, whereas the Vpu proteins of the rare or non-pandemic group N, O and P viruses are partly or completely lacking these functions^19^,^21^,^22^. In this study, we investigated whether CD1d down-regulation and inhibition of iNKT cell activation are conserved functions of Vpu proteins from the four HIV-1 groups and their simian counterparts. Moreover, we mapped amino acid residues required for Vpu-mediated CD1d interference.

## Results

### CD1d down-regulation is a conserved function of primate lentiviral Vpu proteins

The finding that the Vpu proteins of the HIV-1 laboratory strains BaL and NL4-3 (both HIV-1 group M subtype B) down-regulate CD1d from the surface of infected DCs and 293T cells stably expressing CD1d^10^, led us to investigate whether CD1d inhibition is a conserved Vpu function. We analyzed a comprehensive set of 63 *vpu* alleles covering all four HIV-1 groups and related *vpu*-encoding SIV strains (Supplementary Table S1). Our panel included Vpu proteins of 32 HIV-1 group M strains, 10 group O strains, 6 group N strains, and 2 group P strains. The group M Vpu proteins encompassed all 9 subtypes. We also analyzed Vpu proteins of SIV strains infecting central chimpanzees (*Pan troglodytes troglodytes*, cpz*Ptt*; n = 6), Western lowland gorillas (*Gorilla gorilla gorilla*, gor; n = 1), greater spot-nosed monkeys (*Cercopithecus nictitans*, gsn; n = 2), mona monkeys (*Cercopithecus mona*, mon; n = 1), and mustached monkeys (*Cercopithecus cephus*, mus; n = 3). The capacity of these Vpu proteins to down-regulate CD1d cell surface expression was tested in a transient transfection assay and compared to CD4 down-regulation. 293T cells were co-transfected with vectors co-expressing the respective *vpu* alleles and eGFP (or eGFP alone as control) together with human CD1d or CD4 expression constructs, respectively. CD1d and CD4 surface levels were analyzed by flow cytometry 24 h after transfection^10^,^19^.

**Figure 1:**
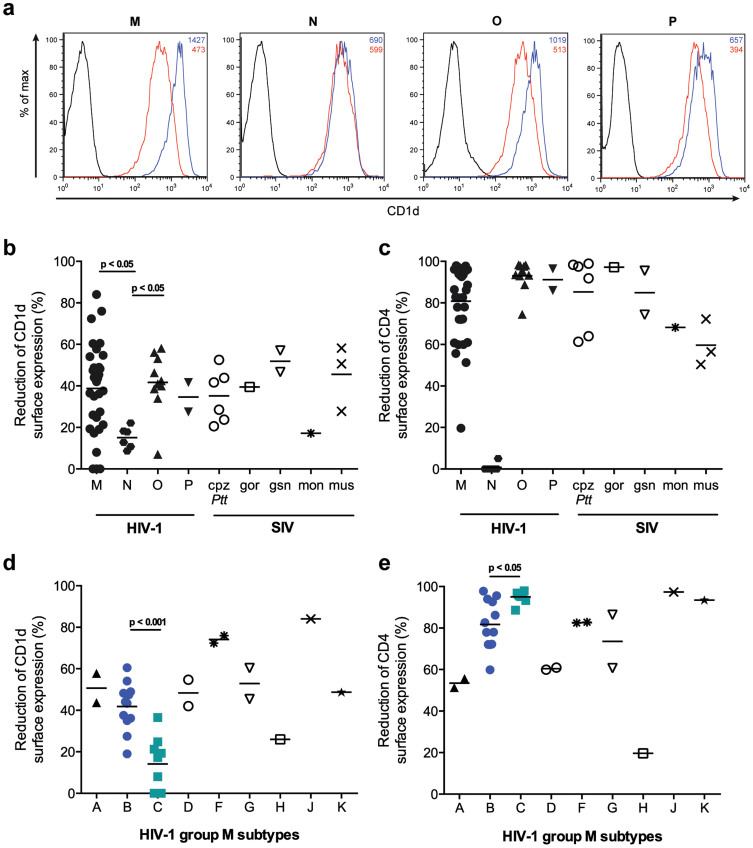
Vpu proteins of HIV-1 groups M, O and P and their SIV precursors down-regulate CD1d. 293T cells were co-transfected with human CD1d or CD4 and *vpu* alleles derived from group M, N, O and P viruses and related SIVs, or eGFP control, respectively. CD1d and CD4 surface expression levels were analyzed 24 h post transfection using flow cytometry. Down-regulation was calculated by comparing CD1d and CD4 surface MFIs in cells expressing Vpu/eGFP and the eGFP control. Each symbol represents one *vpu* allele and the average value of at least three experiments. Horizontal lines indicate average reduction of surface expression of the respective group of *vpu* alleles. (a) Histograms demonstrating the effect of representative HIV-1 group M, N, O and P Vpu proteins on CD1d surface expression. Blue lines indicate cells expressing eGFP control plasmid; red lines, Vpu/eGFP expressing cells; black lines, non-transfected controls. Figures indicate CD1d MFI values in the absence (blue) or presence (red) of Vpu. (b, c) Reduction of CD1d and CD4 surface expression by HIV-1 and SIV Vpu proteins. Statistical analysis was done using GraphPad Prism software and Kruskal-Wallis test with Dunn's multiple comparisons test. (d, e) As in b, c but HIV-1 group M Vpu proteins divided into subtypes are shown. Statistical analysis was done using an unpaired t-test.

In the absence of Vpu, all transfected cells expressed high levels of CD1d and CD4 (Fig. 1a and data not shown). However, co-expression of Vpu proteins of pandemic HIV-1 group M as well as the non-pandemic/rare group O and P viruses resulted in up to 80% reduction of CD1d surface expression (Fig. 1a, b). In contrast, the six *vpu* alleles derived from rare HIV-1 group N strains showed only weak CD1d down-regulation (less than 20%; Fig. 1a, b) confirming previous results^19^. As expected from other studies^21^,^22^, HIV-1 group M, O and P Vpu proteins mediated strong down-regulation of CD4, while those from HIV-1 group N failed to do so (Fig. 1c). Group M Vpu proteins showed a large variation in CD1d down-regulation ranging from 0 to 80%, and separation into subtypes revealed that alleles weakly down-regulating CD1d were found in subtypes H, C and to some extent B (Fig. 1d). All other subtypes, including most B *vpu* alleles, reduced CD1d surface expression by 50% or more. Notably, whereas the panel of tested subtype B Vpu proteins had a significantly stronger capacity to down-regulate CD1d than subtype C Vpu proteins (p <0.001; Fig. 1d), subtype C Vpu proteins were more effective in down-regulating CD4 (p <0.05; Fig. 1e), suggesting that down-regulation of CD1d and CD4 are separable Vpu functions following distinct mechanistic pathways. This was further supported by the finding that the capacities of group M Vpu proteins to down-regulate CD1d and CD4 did not correlate (p = 0.55; Supplementary Fig. S1). These results demonstrate that interference with CD1d surface expression is conserved between most HIV-1 group M, O and P Vpus, but largely lacking in Vpu proteins of rare group N viruses.

Host-specific adaptation of Vpu functions has been observed for tetherin but not for CD4^22^, and next we analyzed the activity of SIV-derived Vpu proteins against human CD1d. We found that *vpu* alleles of SIVcpz*Ptt*, SIVgor, SIVgsn and SIVmus down-regulated human CD1d and CD4 as efficiently as HIV-1 group M *vpu* alleles (Fig. 1b, c). In contrast, the single SIVmon Vpu investigated down-regulated CD4 but had only low activity against human CD1d. Thus, the Vpu proteins of the direct SIV precursors of HIV-1 are active against human CD1d, suggesting that Vpu-CD1d interaction has been conserved and CD1d-mediated immune selection pressure has been important during zoonotic transmission of SIVcpz*Ptt* and SIVgor to humans.

### CD1d down-regulation correlates with decreased iNKT cell activation

**Figure 2:**
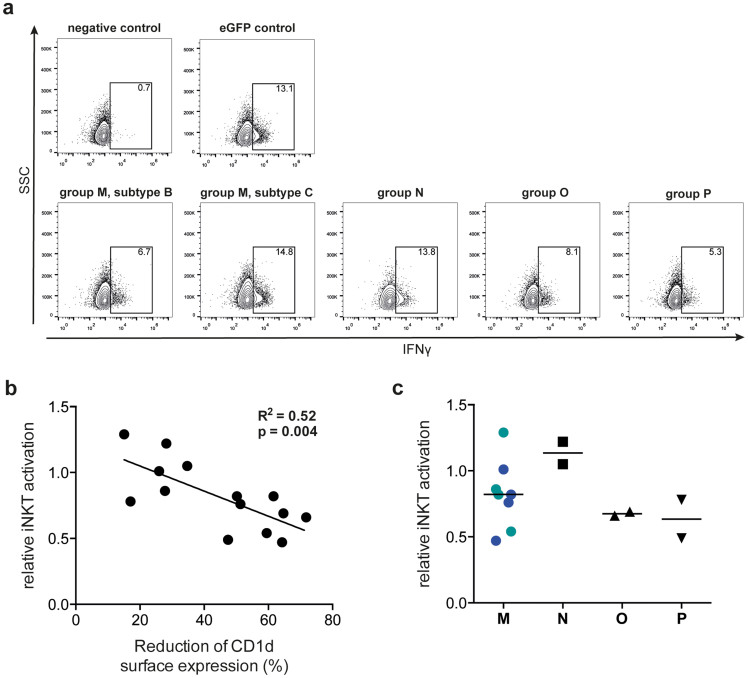
Vpu-mediated down-regulation of CD1d correlates with decreased activation of CD1d-restricted iNKT cells. 293T cells were co-transfected with CD1d and HIV-1 *vpu* alleles or eGFP control, respectively. At 24 h post transfection, cells were used for analysis of CD1d surface expression levels using flow cytometry and loading with the model lipid antigen αGalCer. The human CD4^+^ iNKT cell clone HDD3 was co-incubated with the αGalCer-loaded 293T cells in the presence of brefeldin A for 6 h, and subsequently analyzed for IFN-γ production using flow cytometry. iNKT cell IFN-γ production induced by 293T cells co-transfected with CD1d and eGFP control was set to 1, and all samples were calculated relative to control. (a) Representative dot plots showing iNKT cell IFN-γ production induced by 293T cells co-transfected with CD1d and *vpu* alleles of the indicated HIV-1 groups and controls. (b) Relationship between Vpu-mediated CD1d down-regulation and iNKT cell activation was assessed using linear regression and Pearson correlation. (c) Effect of expression of different HIV-1 *vpu* alleles on IFN-γ production by iNKT cells. Blue and green symbols represent subtype B and C Vpu proteins, respectively. Each data point represents one *vpu* allele and the average value of two experiments performed in at least duplicate.

The different degrees of CD1d down-regulation induced by different Vpu proteins may result in differential inhibition of iNKT cell activation. To test this, 293T cells were transfected with vectors expressing selected Vpu proteins together with human CD1d. Vpu proteins were selected to represent HIV-1 groups M, N, O and P, as well as the variation in CD1d down-regulation induced by different Vpus (Fig. 1b). At 24 h after transfection, CD1d surface expression was analyzed and cells were loaded with the model lipid antigen α-galactosylceramide (αGalCer). αGalCer-loaded cells were co-cultured with the human iNKT cell clone HDD3^23^, and iNKT cell activation was measured by intracellular IFN-γ staining and flow cytometry (Fig. 2a). CD1d down-regulation by the selected Vpu proteins correlated inversely with iNKT cell activation (p <0.01; Fig. 2b). Consequently, the weakly active group N Vpu proteins did not affect iNKT cell activation whereas group M, O and P Vpu proteins reduced both CD1d surface expression and iNKT cell activation. The variation seen in group M reflects the selection of subtype B and C *vpu* alleles differing in their activity against CD1d (Fig. 2c; blue and green symbols represent subtype B and C *vpu* alleles, respectively). These data indicate that the degree of iNKT cell activation is a function of the level of CD1d surface expression and imply that even moderate changes in CD1d expression levels induced by Vpu have significant functional consequences.

### Structural determinants of Vpu-mediated CD1d down-regulation

Vpu has a modular domain structure and consists of an N-terminal TMD and two cytoplasmic α-helices linked by a flexible loop containing two conserved phosphorylation sites^16^. To investigate the possible involvement of TMD interactions in CD1d down-regulation, we tested the NL4-3 mutants VpuRD and VpuΔTMD, harboring a TMD with a scrambled amino acid sequence^17^ or lacking the TMD, respectively (Supplementary Fig. S2). VpuRD mediates degradation of CD4 but has an impaired ability to interfere with tetherin, NTB-A and PVR^5^,^7^,^8^,^17^. Parental Vpu and VpuRD down-regulated CD1d to similar levels, suggesting that sequence integrity of the TMD is not required for this Vpu function. However, membrane anchoring of Vpu is crucial as the Vpu mutant lacking the TMD failed to down-regulate CD1d (Supplementary Fig. S2).

Previous studies have shown that critical determinants for interference with CD4, tetherin and NTB-A localize to the second α-helix of Vpu^24^,^25^,^26^,^27^. Therefore, we next investigated the role of this domain in CD1d down-regulation. Here, we made use of a panel of overlapping triple-alanine mutants scanning through the second α-helix of NL4-3 Vpu (Supplementary Fig. S3). All mutants were expressed together with CD1d in 293T cells, and CD1d surface expression was analyzed in comparison to parental NL4-3 Vpu. Notably, none of these triple-alanine substitutions in the second Vpu α-helix impaired the effect on CD1d (Supplementary Fig. S3). The second α-helix of Vpu contains a putative E_59_XXXL_63_V_64_ trafficking motif that is involved in tetherin antagonism by inducing ESCRT-dependent degradation as well as adapter protein complex 1 (AP1)-dependent mistrafficking^26^,^28^. Interestingly, this motif is conserved in Vpu proteins of HIV-1 group M subtype B but not subtype C strains which only poorly down-regulate CD1d. Mutation of this motif (VpuELV), however, did not abrogate the ability of Vpu to interfere with CD1d surface expression (Supplementary Fig. S3).

**Figure 3:**
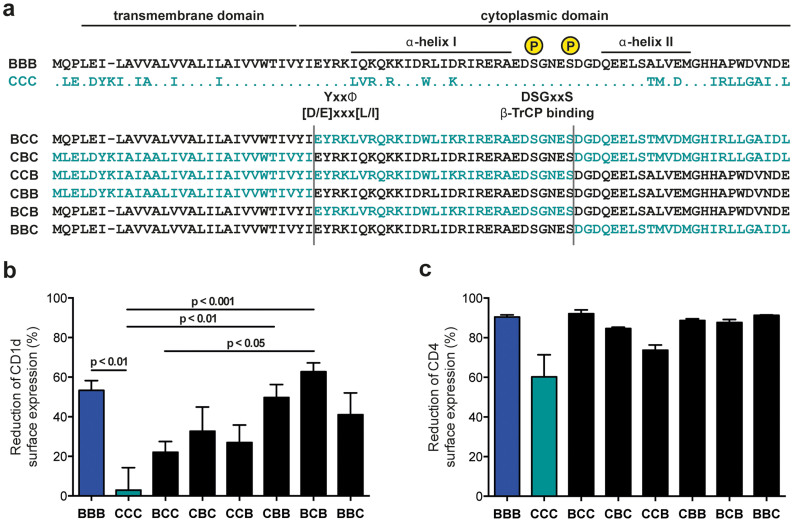
CD1d down-regulation by chimeras between HIV-1 group M subtype B and C Vpu proteins. (a) Schematic representation of WITO.c (BBB; group M subtype B) and ZM247F (CCC; group M subtype C) Vpu chimeras. The TMD, the cytoplasmic domain, α-helical domains 1 and 2, the overlapping putative YxxΦ and [D/E]xxx[L/I] sorting motifs, and the DSGxxS β-TrCP binding site are highlighted. x and Φ correspond to variable and hydrophobic amino acids, respectively. (b, c) 293T cells were co-transfected with CD1d or CD4 and the indicated parental or chimeric Vpu constructs, or eGFP control, respectively. CD1d and CD4 cell surface expression was analyzed by flow cytometry 24 h post transfection, and down-regulation calculated. Average values from at least 3 independent experiments performed in duplicates (±SEM) are shown. Statistical analysis was done using GraphPad Prism software and one-way ANOVA with Tukey's multiple comparisons test.

To assess the involvement of the different Vpu domains in CD1d down-regulation in a broader approach, we took advantage of the functional difference observed between Vpu proteins derived from group M subtype B and C viruses (Fig. 1d). We generated a set of chimeras where the TMDs and regions containing the cytoplasmic α-helices I and II of the Vpu proteins derived from the subtype B virus WITO (BBB) and the subtype C virus ZM247F (CCC) were sequentially exchanged for one another (Fig. 3a). WITO and ZM247F Vpu proteins were selected because they represent particularly strong and weak CD1d down-regulators of the respective subtypes. Analysis of the CD1d down-regulation levels mediated by the different B/C chimeras revealed that the effect of Vpu on CD1d could not be ascribed to the activity of a single Vpu domain. Introduction of any single subtype B Vpu domain into the subtype C Vpu background increased CD1d down-regulation activity compared to the parental C Vpu (CCC) but none of the B Vpu domains was sufficient to rescue activity to the level of the parental B Vpu (BBB) (Fig. 3b). Likewise, all reciprocal chimeras efficiently interfered with CD1d, indicating that no single Vpu C domain has a dominant negative impact on CD1d down-regulation. However, we observed statistically significant differences between chimera BCB and both the parental C Vpu (CCC) and chimera BCC (p <0.001 and 0.05, respectively; Fig. 3b), suggesting that the C-terminal part of Vpu contains determinants necessary for efficient CD1d inhibition. Functional expression of all B/C chimeras was confirmed by CD4 down-regulation (Fig. 3c) and Western blot analysis (Supplementary Fig. S4).

### Identification of a conserved APW motif at the C-terminus of subtype B Vpu proteins involved in CD1d down-regulation

**Figure 4:**
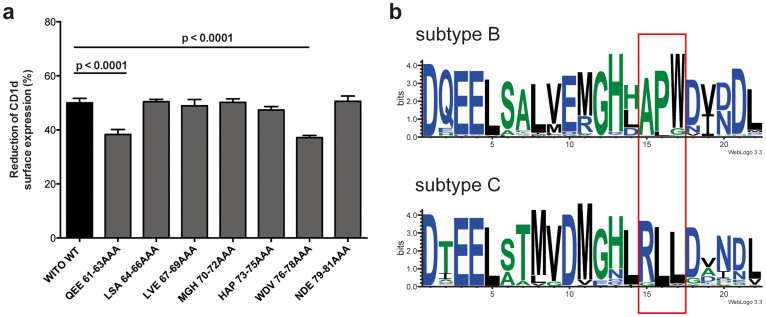
Determinants at the C-terminus of Vpu contribute to CD1d down-regulation. (a) 293T-CD1d cells were transfected with the indicated non-overlapping triple-alanine mutants of the WITO Vpu C-terminus. 24 h post transfection, cells were surface stained with anti-CD1d antibody and analyzed by flow cytometry. Average values from at least 3 independent experiments preformed in duplicates (±SD) are shown. Statistical analysis was done using GraphPad Prism software and one-way ANOVA with Dunnett's multiple comparisons test. (b) Vpu amino acid sequences of subtype B (n > 5500) and subtype C (n > 3000) were retrieved from the HIV sequence database (www.hiv.lanl.gov) and the C-terminal 22 residues of the sequences analyzed using WebLogo 3.0 software (weblogo.threeplusone.com/create.cgi). Colour coding of the amino acids represents hydrophobicity; blue, hydrophilic; green, neutral; black, hydrophobic. Conserved motifs are highlighted.

The findings that the second α-helix of Vpu appears to not be directly involved in CD1d down-regulation (Supplementary Fig. S3), and that the C-terminal third of Vpu may contribute to the overall efficiency of CD1d inhibition (Fig. 3b), led us to speculate that C-terminal amino acids outside the second α-helix may play a role. To address this, we analyzed CD1d down-regulation by non-overlapping triple-alanine mutants of the WITO Vpu C-terminus including the second α-helix (Supplementary Fig. S5). We found that exchange of amino acid residues 61–63 (QEE) located at the N-terminal end of the second α-helix and 76-78 (WDV) located C-terminally of the second α-helix resulted in moderate but significant reductions in CD1d down-regulation activity (p <0.0001; Fig. 4a). To verify the potential relevance of these residues, we performed a comprehensive sequence alignment of the 22 C-terminal amino acids of more than 5500 subtype B and 3000 subtype C Vpu sequences available from the HIV sequence data base (www.hiv.lanl.gov). LogoPlot analyses revealed conservation of residues 61 and 76 (WITO annotation) within but not between subtype B and C Vpu proteins (Q61/W76 vs. T61/L76; Fig. 4b). Considering the functional difference between these subtypes, this may indicate a role for residues 61 and 76 in CD1d down-regulation. However, as the WITO (subtype B) and ZM247F (subtype C) Vpu proteins differ in their abilities to interfere with CD1d but both carry Q61 (Fig. 3), this amino acid position probably has minor importance for CD1d down-regulation. Notably, W76 and L76 are part of conserved APW and RLL motifs at position −8 to −6 from the C-terminus highly specific for subtype B and C Vpus, respectively (Fig. 4b). The (L)RLL motif in subtype C Vpu proteins was recognized in earlier studies and suggested as a signal targeting Vpu to the cell membrane^27^,^29^,^30^.

**Figure 5:**
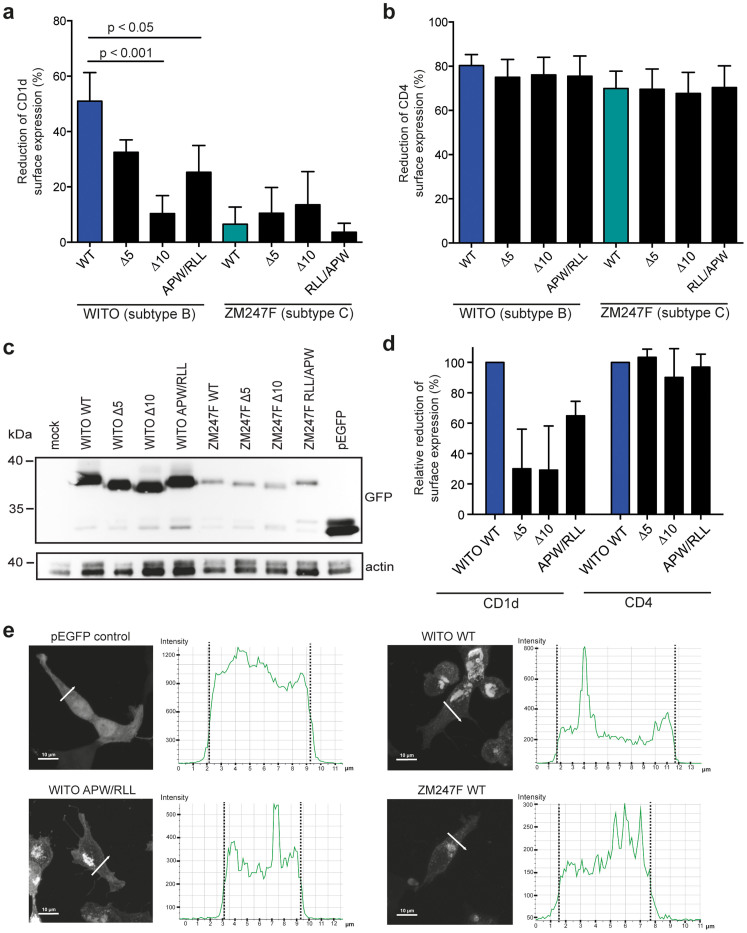
The conserved APW motif of subtype B Vpu proteins is involved in CD1d down-regulation. (a, b) 293T cells were co-transfected with CD1d or CD4 and the indicated WITO and ZM247F Vpu deletion and amino acid exchange mutants, respectively. CD1d and CD4 cell surface expression was analyzed by flow cytometry 24 h post transfection, and down-regulation calculated. Average values from at least 3 independent experiments preformed in duplicates (±SD) are shown. Statistical analysis was done using a Kruskal-Wallis test with Dunn's multiple comparisons test. (c) Expression analysis of parental and mutant Vpu proteins by Western blot. 293T cells were transfected with the indicated eGFP-tagged Vpu proteins, and 24 h post transfection cell lysates were prepared, separated on a 12% SDS–polyacrylamide gel electrophoresis gel and transferred onto a nitrocellulose membrane. The membrane was probed with anti-GFP and anti-actin antibodies. Blots have been cropped; for full-size uncropped blots see Supplementary Fig S6. (d) Human monocyte-derived dendritic cells were infected with the indicated VSV-G pseudotyped HIV-1 viruses. 7 days post infection, cells were surface stained with anti-CD1d and anti-CD4 antibodies followed by permeabilization, anti-p24 staining and flow cytometry analysis. Down-regulation was calculated by comparing CD1d and CD4 MFIs on p24 negative and positive cells; down-regulation by WITO WT was set to 100%. Data show experiments with MDDCs generated from 3 different donors. Error bars represent SD. (e) 293T cells were transfected with the indicated eGFP-tagged Vpu constructs or eGFP control, respectively. 24 h post transfection, cells were fixed and examined by fluorescence microscopy. eGFP signal intensities were measured along the indicated lines traversing the cells using NIS-Elements A1R software. Vertical dashed lines in the intensity plots indicate cell edges. For all images, contrast and brightness were changed for visualization purposes throughout the entire image. Images shown are representative of 2 independent experiments. Scale bars, 10 μm.

To test the involvement of the APW and RLL motifs in Vpu function, we constructed WITO and ZM247F Vpu mutants lacking 5 or 10 C-terminal amino acids and specific mutants where these motifs were swapped (Δ5, Δ10 and APW/RLL alt. RLL/APW; Supplementary Fig. S6). The deletion of 5 amino acids from the WITO Vpu C-terminus (WITOΔ5) had a moderate though not statistically significant effect on CD1d down-regulation, whereas the WITOΔ10 mutant almost completely lost its down-regulation activity (p <0.001; Fig. 5a). This indicated an important determinant between residues −5 and −10, and indeed, the APW/RLL exchange significantly reduced the CD1d down-regulation activity of WITO Vpu (p <0.05). In contrast, ZM247F wild-type and all mutants tested were poor CD1d antagonists (Fig. 5a). These data show that the APW motif is necessary (in the subtype B Vpu context) but not sufficient (in the subtype C Vpu context) for CD1d interference. Of note, all mutant Vpu proteins efficiently reduced CD4 surface expression showing that the 10 C-terminal amino acids of subtype B and C Vpus are not involved in this Vpu function (Fig. 5b). Western blot analysis further confirmed the expression of all constructs (Fig. 5c).

Next, we wanted to verify the significance of the identified C-terminal Vpu motifs for CD1d down-regulation by infecting human monocyte-derived dendritic cells with HIV-1 NL4-3 expressing wild-type WITO Vpu or WITO Vpu mutants Δ5, Δ10, and APW/RLL respectively. All viruses were *nef*-defective to eliminate any effect of the Nef protein on CD1d expression^9^,^10^,^31^. Whereas all mutant viruses had a reduced ability to down-regulate CD1d as compared to wild-type, none of the mutations affected CD4 down-regulation (Fig. 5d). Taken together, the above results demonstrate the existence of a highly conserved APW motif C-terminal of the second α-helix in subtype B Vpu proteins that is necessary for CD1d interference. However, the APW motif is not sufficient to rescue CD1d down-regulation activity in otherwise poorly active subtype C Vpu proteins.

Previous studies have suggested that subtype B Vpu proteins are retained in the Golgi apparatus whereas subtype C Vpu proteins localize also to the plasma membrane. This difference has been attributed to the presence and absence of Golgi retention signals in the cytoplasmic domain of Vpu^27^,^32^,^33^. To determine whether the APW motif in subtype B Vpu proteins functions as a sorting signal, we analyzed the subcellular distribution of eGFP-tagged WITO, WITO APW/RLL or ZM247F Vpu constructs 293T cells using fluorescence microscopy. All Vpu constructs showed similar subcellular distributions with the majority of Vpu localized to the Golgi region and intracellular vesicles (Fig. 5e, micrographs). Analyses of Vpu signal intensities throughout the cell did not indicate any significant membrane accumulation (Fig. 5e, histograms). These results suggest that differences in subcellular distribution may not explain the functional differences between subtype B and C Vpu proteins, and moreover, that the APW motif necessary for CD1d down-regulation by subtype B Vpu proteins does not represent a major sorting signal.

## Discussion

The *vpu* open-reading frame is found in HIV-1 and some related SIV strains but absent from the genomes of HIV-2 and most SIV strains. Vpu is a multifunctional accessory protein and differences in its activities have been implicated in the efficiency of virus spread and pathogenesis *in vivo*^34^. In the present study, we have investigated the ability of a comprehensive set of primate lentiviral Vpu proteins to interfere with CD1d-mediated immunity. We found that CD1d down-regulation is a conserved function of Vpu proteins from HIV-1 groups M, O and P as well as their direct precursors SIVcpz*Ptt* and SIVgor. In contrast, Vpu proteins of rare group N viruses largely lack the CD1d down-regulation function. At the group M subtype level, subtype C Vpu proteins were significantly weaker CD1d antagonists than subtype B Vpu proteins. The physiological relevance of these findings is highlighted by the observation that CD1d down-regulation mediated by the different Vpu proteins correlated inversely with the levels of iNKT cell activation. CD1d-restricted iNKT cells are rapid and potent producers of cytokines and chemokines linking the innate and adaptive immune responses^35^,^36^. Therefore, it is tempting to speculate that evasion from iNKT cell recognition, in particular during the earliest stages of infection at mucosal sites prior to initiation of antigen-specific immune responses, may contribute to the establishment of infection and viral spread. In humanized mouse models it has been shown that Vpu is a critical factor for early viral dissemination, however, the impact of Vpu on iNKT cell responses in such models has not been investigated yet^37^,^38^,^39^. Further studies should evaluate the relative importance of CD1d interference in the context of other known Vpu functions in primary cells. Noteworthy, the experimental model system employed here used a CD1d over-expression system and the strongly activating model lipid antigen αGalCer. *In vivo*, CD1d surface expression levels on antigen-presenting cells are generally low and iNKT cell activation induced by endogenous lipid antigens, such as GlcCer, may be relatively weak despite the presence of cytokines and other co-stimulating factors^12^,^13^. Therefore, the model system employed here may underestimate the effect of Vpu.

There is accumulating evidence that HIV-1 subtype B and C Vpu proteins differ in their functional properties. Whereas B Vpus degrade CD4 efficiently and interfere with tetherin expression and virus particle release, some studies have shown that C Vpus are less potent in these functions although the number of Vpu proteins analyzed was limited^32^,^40^,^41^. In this study, comparing 12 subtype B and 9 subtype C Vpu proteins, we found that subtype C Vpus were significantly weaker CD1d antagonists than their subtype B counterparts (Fig. 1d). In contrast, subtype C Vpu proteins were more efficient in down-regulating CD4. It is important to note, however, that even B Vpus reduced CD4 surface expression on average by 80% and it remains to be determined if this difference is physiologically relevant (Fig. 1e). Recently, it has been reported that subtype B Nef proteins have a stronger ability to down-regulate CD4 and HLA class I than subtype C Nefs, indicating that subtype B/C differences in functionality of the HIV-1 accessory proteins are not limited to Vpu and may even influence each other^42^. Considering the high global prevalence of subtype B and C viruses, these differences are striking. Some studies have suggested that subtype C viruses have lower replicative fitness *in vitro*^43^,^44^,^45^, but clear evidence for differences in disease progression between subtype B and C infections is lacking. Hence, further studies need to address potential consequences of the functional differences between Vpu and other accessory proteins derived from different HIV-1 group M subtypes.

The SIVs of central chimpanzees and Western lowland gorillas have crossed the species barrier from apes to humans and gave rise to the four known HIV-1 groups M, N, O, and P^20^. However, only group M viruses have spread globally and caused the AIDS pandemic. It is believed that adaptation to effectively evade intrinsic, innate and adaptive anti-viral defence mechanisms of the human host, as shown for Vpu-mediated tetherin antagonism, is one reason for the success of these viruses^19^,^22^,^34^. We found that already the Vpu proteins of the HIV-1 precursors SIVcpz*Ptt* and SIVgor interfere with human CD1d expression (Fig. 1b). In contrast to tetherin antagonism, our data suggest pre-existing susceptibility of human CD1d to SIVcpz*Ptt* and SIVgor Vpu proteins rather than host-specific adaptation of these Vpus to inhibit human CD1d. This may not be surprising considering the high degree of conservation between human, chimpanzee and gorilla CD1d. After transmission to humans and during the subsequent adaptation process, most Vpu proteins of pandemic group M viruses, including subtypes A, B and D, seem to have maintained the ability to interfere with human CD1d and CD4 and gained anti-tetherin activity. In contrast, the Vpu proteins of group M subtype C viruses have partly lost the CD1d down-regulation function and gained anti-tetherin activity. This supports the possibility that Vpu's ability to down-regulate human CD1d may have contributed to successful cross-species transmission. It is important to note that viruses lacking Vpu-mediated CD1d down-regulation may not be devoid of this function as they may use other means to inhibit CD1d. There is evidence that CD1d down-regulation is a function of both Vpu and Nef, and CD1d down-regulation has been demonstrated for HIV-1 group M Nef proteins^9^,^10^,^31^ though not in detail on the subtype level. Thus, pandemic group M viruses may employ two accessory proteins, expressed early (Nef) and late (Vpu) during the viral replication cycle, to inhibit recognition by CD1d-restricted iNKT cells emphasizing the importance of this immune evasion mechanism. It is so far unknown if the Nef proteins of HIV-1 group M subtype C and group N, O, and P strains down-regulate CD1d. Thus, further studies should aim to elucidate if Nef proteins of primate lentiviruses that express Vpu proteins lacking anti-CD1d activity compensate for this functional deficiency.

Vpu employs distinct mechanisms to interfere with its cellular targets. Firstly, it regulates the stability of CD4 and tetherin by promoting their ubiquitination and subsequent degradation. For this, Vpu acts as a viral adaptor protein linking the E3 ubiquitin ligase complex substrate adaptor β-TrCP to the cytoplasmic domains of its target proteins (reviewed in Ref. 16). The interaction of Vpu and β-TrCP is well-defined and requires the phosphorylation of Vpu at two highly conserved serine residues located between the first and second cytoplasmic α-helix. Secondly, Vpu interferes with intracellular protein trafficking; it affects the anterograde transport of NTB-A^24^ and inhibits the recycling of CD1d and tetherin from endosomal compartments to the cell surface^10^,^46^. The molecular mechanisms employed by Vpu to interfere with the trafficking of host cell proteins are largely unknown. In general, the accessory proteins of primate lentiviruses act as adaptors linking their targets to one or more cellular co-factors involved in ubiquitination or trafficking pathways^47^. It is therefore possible that Vpu binds to adaptor-protein complexes (APs) or other cellular co-factors involved in subcellular trafficking pathways. Although the Vpu cytoplasmic domain contains classical tyrosine and leucine-based binding motifs, such interactions were difficult to demonstrate in the past^48^. In a recent study, Jia et al. reported interaction of Vpu with multiple subunits of AP1 and AP2^28^. Binding of Vpu to AP1 involved the E_59_XXXL_63_V_64_ (ELV) motif and was shown to be important for Vpu-mediated tetherin antagonism. Notably, in our experiments mutation of the ELV motif did not affect CD1d down-regulation (Supplementary Fig. S3), suggesting that AP1-dependent trafficking pathways are not involved in Vpu-mediated CD1d interference or motifs other than ELV mediate Vpu binding to AP1. More studies are needed to determine if Vpu-mediated mistrafficking of CD1d, NTB-A and PVR follows similar molecular pathways and involves common cellular co-factors.

The interaction between Vpu and its target proteins involves multiple Vpu domains. Whereas Vpu and CD4 interact directly through their cytoplasmic domains^17^, TMD interactions were shown to be important for tetherin, NTB-A and PVR^5^,^7^,^8^. In contrast to the latter molecules, CD1d down-regulation is unaffected by scrambling the Vpu TMD. Although it cannot be excluded that single amino acid positions in the TMD still play a role in CD1d down-regulation, our data suggest interaction between the cytoplasmic portions of Vpu and CD1d rather than TMD interactions. A set of chimeras based on the active subtype B Vpu from the WITO isolate and the largely inactive subtype C Vpu from the ZM247F isolate revealed that no single domain determines the ability or inability of Vpu to down-regulate CD1d. This suggests that binding surfaces formed by different parts of the Vpu cytoplasmic domain rather than linear motifs are involved in the interaction with CD1d. Nonetheless, combining mutational and sequence analysis approaches, we identified a highly conserved APW motif at the C-terminus of subtype B Vpu proteins that is necessary but not sufficient for CD1d down-regulation (Fig. 4 and 5). Notably, C-terminal deletion mutants lacking the APW motif are fully capable of down-regulating CD4 and antagonizing tetherin (Fig. 5b, d and^27^,^49^). To our knowledge, this is the first time the APW motif has been implicated in Vpu function. As the APW motif is specific for HIV-1 group M subtype B and the closely related subtype D Vpu proteins, further studies will need to address what motifs are involved in CD1d down-regulation mediated by Vpu proteins lacking this sequence. Interestingly, a recent paper by Jafari et al. described a W76G polymorphism in primary subtype B Vpu clones and identified W76 as important for enhancement of virus particle release without affecting surface expression of tetherin^50^. However, in our experiments the single W76G exchange, as well as other single exchanges in the TMD and at the C-terminus (Supplementary Fig. S7), did not alter the ability of Vpu to down-regulate CD1d or CD4 (Supplementary Fig. S7). This may seem contradictory but structural data derived from the Vpu cytoplasmic domain of HIV-1 HV1S1 (group M subtype B) suggest that the C-terminal residues L73-V78, encompassing the APW motif, form a stable loop with a hydrogen bond connecting A74 and V78, and W76 located at the tip of the loop^51^,^52^. More experimental work will be required to demonstrate involvement of the C-terminal loop structure in CD1d downregulation, but we speculate that residues outside the subtype B-specific APW motif may be involved in CD1d interference by contributing to the loop formation. The unexpected anti-CD1d activity of some Vpu mutants used in this study, including mutants HAP73-75AAA (Fig. 4a) and W76G (Supplementary Fig. S7), may possibly be explained by their ability to maintain the described loop structure.

## Methods

### Expression constructs and mutagenesis

Vpu proteins were expressed without or with AU-1 tag from the pCG vector co-expressing eGFP via an IRES or expressed as eGFP fusion proteins from the vector pEGFP-N3^10^,^19^,^21^,^22^. Human *CD1d* and *CD4* genes were cloned into the vector pcDNA3.1/Zeo(+) (Invitrogen). NL4-3, WITO and ZM247F Vpu mutants were generated by PCR-based standard methods using primers containing the desired mutations or overlapping primers (Supplementary Fig. S5 and S6). Vpu B/C chimeras were synthesized by Eurofins and cloned into the pEGFP-N3 vector. Proviral NL4-3 vectors expressing wild-type WITO Vpu or WITO Vpu mutants APW/RLL, Δ5 and Δ10, respectively, were generated by cloning the respective *vpu* genes into the SacII and NcoI restriction sites in pBR-NL4-3 Δ*vpu*IRES*env*Δ*nef*. The cloning procedure eliminated the IRES between the *vpu* and *env* genes requiring VSV-G pseudotyping of the viruses to ensure infectivity. All expression vectors were confirmed by sequence analysis.

### Cell culture, transfection and production of virus stocks

293T cells were cultured in Dulbecco's modified Eagle medium (GIBCO Invitrogen Corporation) supplemented with 10% fetal calf serum, 2 mM L-glutamine and antibiotics. Cells were transiently transfected with DNA constructs using Lipofectamine 2000 (Invitrogen) or Turbofect (Fermentas) according to the manufacturer's protocols and 24 h post transfection analyzed in the respective assays. To produce stocks of VSV-G pseudotyped virus, 293T cells were co-transfected with proviral DNA and pVPack VSV-G plasmid (Stratagene). At 48 h post transfection, virus containing cell culture supernatants were harvested, cleared and frozen.

### Flow cytometry and surface protein down-regulation

Surface and intracellular flow cytometry were performed according to standard procedures^53^. CD1d was detected with mouse anti-human CD1d (clone CD1d42) and goat anti-mouse Ig conjugated to APC or anti-CD1d-PE (clone CD1d42), CD4 with anti-CD4-APC (clone SK3). All antibodies were from BD Biosciences. Percent reduction of protein surface expression was calculated by comparing CD1d and CD4 mean fluorescence intensities (MFI) in control cells expressing eGFP alone (MFI^contr^) versus sample cells expressing eGFP together with Vpu (MFI^sample^) in the respective experiments ((MFI^contr^ - MFI^sample^)/MFI^contr^) × 100. Data were acquired on FACSCalibur or LSRFortessa flow cytometers (BD Biosciences) and analyzed using FlowJo Version 9 software (Tree Star).

### iNKT cell activation assay

293T cells were co-transfected with Vpu and CD1d expression vectors and 24 h post transfection loaded with 100 ng/mL αGalCer (KRN7000; Biomol International) for 2 h. 293T cells were then co-incubated with the human CD4^+^ iNKT cell clone HDD3^23^ at a 1:2 ratio in the presence of brefeldin A (GolgiPLUG; 2 mg/mL; BD Biosciences) for 6 h. To assess iNKT cell IFN-γ production using flow cytometry, cells were stained with anti-CD3-PerCP (clone SK7; BD Biosciences) followed by saponin permeabilization and intracellular staining with anti-IFN-γ-APC (clone 25723.11; BD Biosciences).

### Western blot

To monitor protein expression, 293T cells were transfected with eGFP-tagged Vpu constructs and 24 h later lysed in buffer containing 1% Triton and Halt Protease Inhibitor Cocktail (Pierce). Cell lysates were separated on 12% Bis-Tris gels (LifeTechnologies), transferred onto nitrocellulose membranes, and probed with HRP-conjugated anti-GFP (Miltenyi) or anti-AU-1 (Thermo Fisher Scientific) followed by HRP-conjugated donkey anti-goat (R&D Systems), and mouse anti-actin (MP Biomedicals) antibodies followed by HRP-conjugated anti-mouse IgG (GE Healthcare Life Sciences).

### Generation and infection of human monocyte-derived dendritic cells

Human monocyte derived-dendritic cells (MDDCs) expressing CD1d were generated and infected as described^54^,^55^. Briefly, anonymous buffy coats from healthy blood donors were purchased from the Karolinska University Laboratory; buffy coats were used in accordance with approval by the ethics committee at Karolinska Institutet. After enrichment with RosetteSep human monocyte enrichment cocktail (StemCell Technologies), monocytes were cultured for 6–7 days in RPMI 1640 medium supplemented with 5% human serum (Sigma-Aldrich), rhIL-4 (6.5 ng/mL; R&D Systems) and rhGM-CSF (250 ng/mL; PeproTech) to obtain immature MDDCs. MDDCs were infected with viral stocks, cultured in the presence of cytokines and serum for 7 days, and finally analyzed for the expression of surface proteins and intracellular HIV-1 p24 by flow cytometry.

### Microscopy

293T cells were transfected with vectors expressing eGFP-tagged Vpu, and 4 h later split onto glass coverslips. 24 h post transfection, cells were fixed with 2% PFA for 20 min at room temperature and mounted on slides using ProLong Anti-Fade Gold with DAPI (LifeTechnologies). Images were obtained on a Nikon A1R confocal system with a 60×/1.49 oil objective and analyzed using NIS-Elements A1R software (Version 3.2; Nikon Instruments Europe).

### Sequence analysis and statistical tests

Amino acid sequences of HIV-1 group M subtype B (n > 5500) and subtype C (n > 3000) Vpu proteins were retrieved from the HIV sequence data base (www.hiv.lanl.gov) and aligned with Jalview Version 2 software (www.jalview.org)^56^. LogoPlots of the 20 C-terminal amino acid residues of all sequences were generated with WebLogo 3.0 software (weblogo.threeplusone.com/create.cgi) using default settings^57^,^58^. Data were analyzed using unpaired two-tailed t-test and Kruskal-Wallis test or one-way ANOVA followed by post tests for multiple comparisons, as appropriate. Correlations were evaluated using linear regression and Spearman or Pearson correlation. All statistical analyses were performed using Prism Version 6 software (GraphPad). P <0.05 was considered statistically significant.

## Author Contributions

S.M.B. participated in the design of the study, performed most of the experiments, analyzed data, and co-wrote the manuscript. D.S. participated in study design and performed cloning experiments. S.S. performed experiments. J.K.S. and F.K. participated in the study design. M.M. participated in study design and data analyses, coordinated the study and wrote the manuscript. All authors read and approved the final manuscript.

## Supplementary Material

Supplementary Information Supplementary Information
