# Supplementary material for: Involvement of a C-terminal motif in the interference of primate lentiviral Vpu proteins with CD1d-mediated antigen presentation
Source: Sci Rep. 2015 Apr 15;5:9675. doi: 10.1038/srep09675 (PMC4397644; doi:10.1038/srep09675)
Supplement: Supplementary Information [file srep09675-s1.pdf]

Supplementary information

Involvement of a C-terminal motif in the interference of primate lentiviral Vpu proteins with CD1d-mediated antigen presentation

Susanna M. Bächle[1], Daniel Sauter[2], Sabrina Sibitz[1], Johan K. Sandberg[1], Frank Kirchhoff[2], Markus Moll[1*]

[1]Center for Infectious Medicine, Department of Medicine, Karolinska Institutet, Karolinska University Hospital Huddinge, 14186 Stockholm, Sweden

[2]Institute of Molecular Virology, Ulm University Medical Center, 89081 Ulm, Germany

*Corresponding author

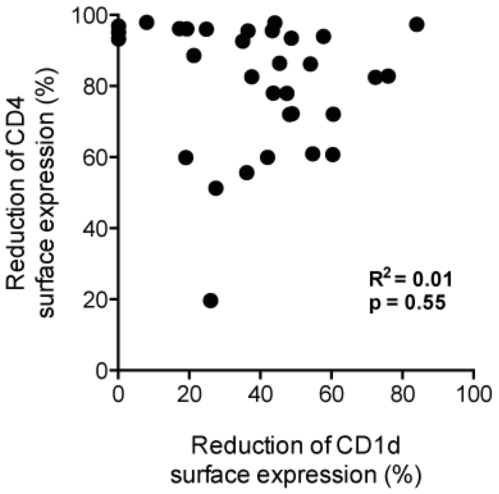

Supplementary Figure S1 Down-regulation of CD1d and CD4 are mechanistically separable Vpu functions.

Correlation between reduction of CD1d and CD4 surface expression induced by HIV-1 group M Vpu proteins was assessed using linear regression and Spearman correlation. Each symbol represents one *vpu* allele and the average value of at least three experiments.

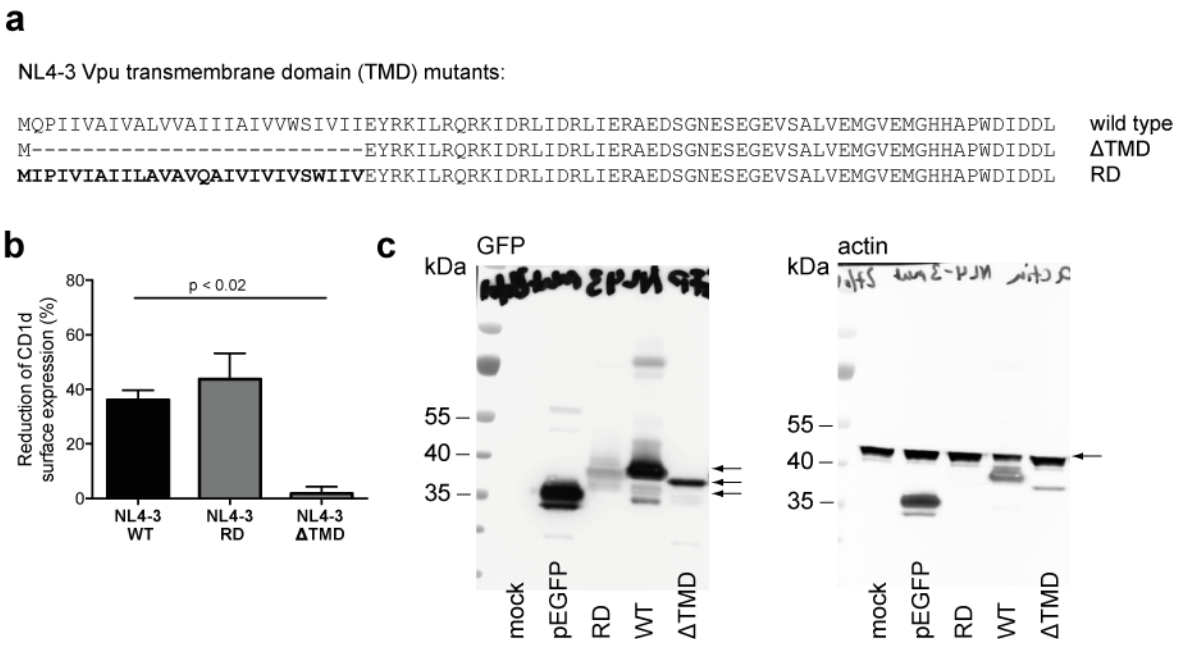

Supplementary Figure S2 CD1d down-regulation by Vpu transmembrane domain mutants.

(a) Amino acid sequences of NL4-3 Vpu and TMD mutants VpuRD and VpuΔTMD; amino acid deletions are indicated by dashes, amino acid exchanges by bold letters. (b) 293T cells were co-transfected with CD1d and eGFP fusion constructs of the indicated Vpu proteins. At 24 h post transfection, CD1d surface expression was analyzed using flow cytometry. Average values from 3 independent experiments preformed in duplicates (±SD) are shown. Statistical significance was assessed using one-way ANOVA with Dunnett's multiple comparisons test. (c) 293T cells were transfected with the indicated eGFP-tagged Vpu proteins. At 24 h post transfection, cell lysates were prepared and analyzed by Western Blot using HRP-conjugated anti-GFP antibody followed by mouse anti-actin antibody and HRP-conjugated anti-mouse IgG. Uncropped full-size blots are shown.

a

NL4-3 Vpu triple-alanine mutants 2.α helix:

```
MQPIIVAIVALVVAIIIAIVVWSIVIIEYRKILRQRKIDRLIDRLIERAEDSGNESEGEVSALVEMGVEMGHHAPWDIDDL   wild type

MQPIIVAIVALVVAIIIAIVVWSIVIIEYRKILRQRKIDRLIDRLIERAEDSGNESEGAAAALVEMGVEMGHHAPWDIDDL   59-61A
MQPIIVAIVALVVAIIIAIVVWSIVIIEYRKILRQRKIDRLIDRLIERAEDSGNESEGEAAALVEMGVEMGHHAPWDIDDL   60-62A
MQPIIVAIVALVVAIIIAIVVWSIVIIEYRKILRQRKIDRLIDRLIERAEDSGNESEGEVAAAVEMGVEMGHHAPWDIDDL   61-63A
MQPIIVAIVALVVAIIIAIVVWSIVIIEYRKILRQRKIDRLIDRLIERAEDSGNESEGEVSAAAEMGVEMGHHAPWDIDDL   62-64A
MQPIIVAIVALVVAIIIAIVVWSIVIIEYRKILRQRKIDRLIDRLIERAEDSGNESEGEVSAAAAMGVEMGHHAPWDIDDL   63-65A
MQPIIVAIVALVVAIIIAIVVWSIVIIEYRKILRQRKIDRLIDRLIERAEDSGNESEGEVSALAAAGVEMGHHAPWDIDDL   64-66A
MQPIIVAIVALVVAIIIAIVVWSIVIIEYRKILRQRKIDRLIDRLIERAEDSGNESEGEVSALVAAAVEMGHHAPWDIDDL   65-67A
MQPIIVAIVALVVAIIIAIVVWSIVIIEYRKILRQRKIDRLIDRLIERAEDSGNESEGEVSALVEAAAEMGHHAPWDIDDL   66-68A
MQPIIVAIVALVVAIIIAIVVWSIVIIEYRKILRQRKIDRLIDRLIERAEDSGNESEGEVSALVEMAAAMGHHAPWDIDDL   67-69A
MQPIIVAIVALVVAIIIAIVVWSIVIIEYRKILRQRKIDRLIDRLIERAEDSGNESEGEVSALVEMGAAAGHHAPWDIDDL   68-70A
MQPIIVAIVALVVAIIIAIVVWSIVIIEYRKILRQRKIDRLIDRLIERAEDSGNESEGAVSAAAEMGVEMGHHAPWDIDDL   ELV
```

b

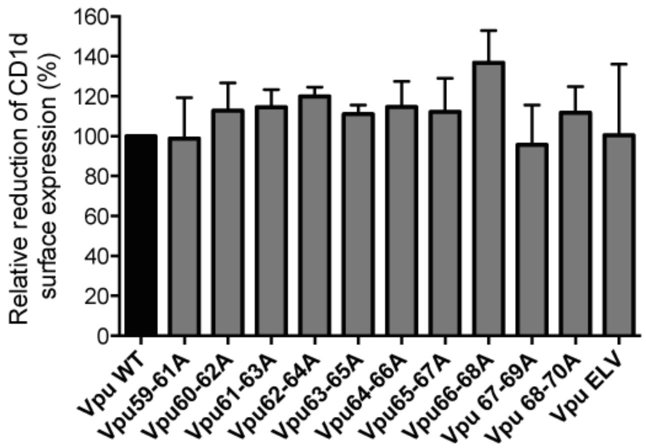

Supplementary Figure S3 Analysis of triple-alanine mutants scanning through the second α-helix of NL4-3 Vpu.

(a) Amino acid sequences of NL4-3 Vpu and overlapping triple-alanine mutants scanning through the second α-helix; bold letters indicate amino acid exchanges. (b) 293T cells were co-transfected with CD1d and the indicated C-terminally HA-tagged NL4-3 Vpu constructs, respectively. 24 h post transfection, cells were surface stained with anti-CD1d antibodies, permeabilized and stained with anti-HA antibody to detect Vpu-expressing cells. CD1d down-regulation by wild-type Vpu (VpuWT) was set to 100%. Average values from at least 3 independent experiments preformed in duplicates (±SD) are shown. Statistical analysis was done using GraphPad Prism software and one-way ANOVA with Dunnett's multiple comparisons test.

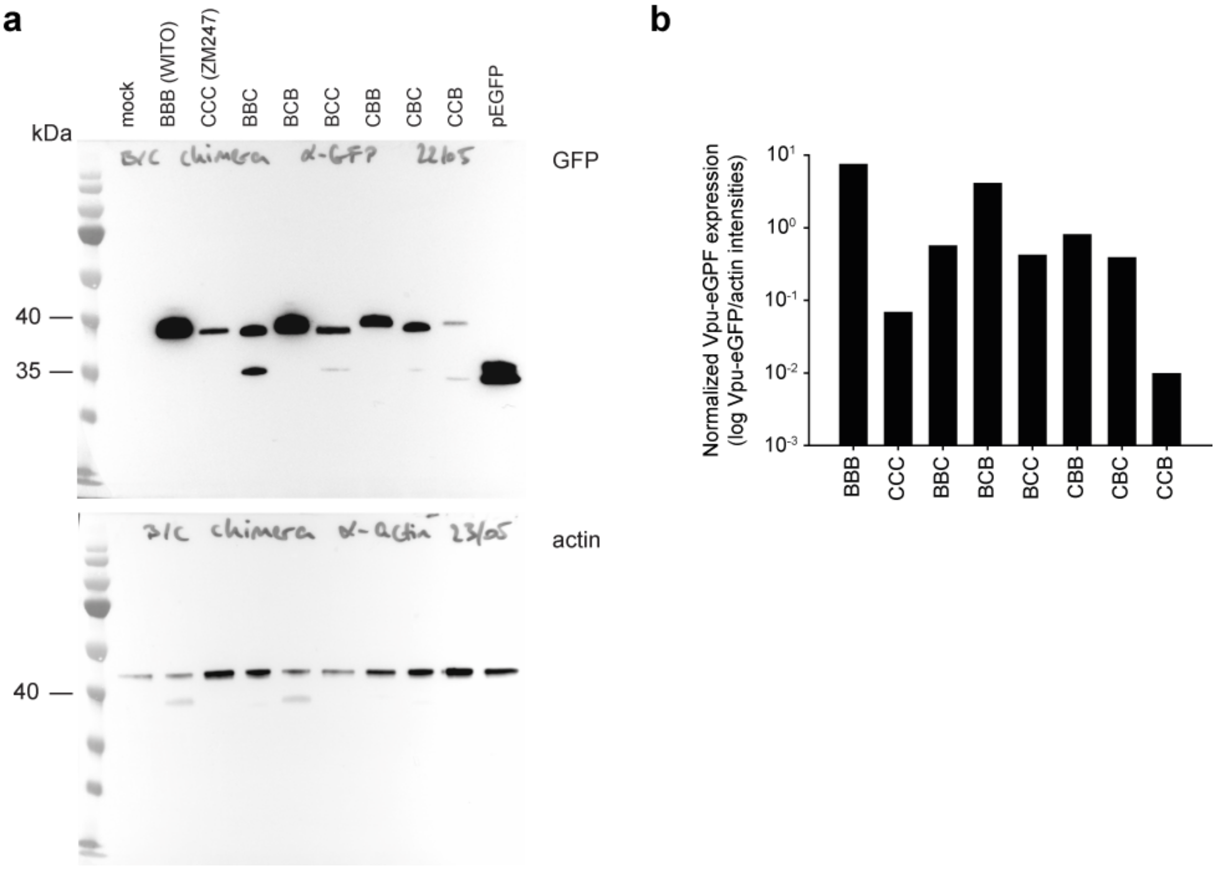

Supplementary Figure S4 Expression of parental subtype B (WITO.c) and C (ZM247F) Vpu proteins and B/C chimeras.

(a) 293T cells were transfected with the indicated eGFP-tagged Vpu proteins. At 24 h post-transfection, cell lysates were prepared and analyzed by Western Blot using HRP-conjugated anti-GFP and mouse anti-actin antibodies followed by HRP-conjugated anti-mouse IgG. Uncropped full-size blots are shown. (b) Vpu expression levels were quantified from the Western blot shown in panel A and normalized to actin.

a

WITO (subtype B) Alanine scan mutants 2.α helix to C-terminus

MQPLEILAVVALVVALILAIVVWTIVYIEYRKIQKQKKIDRLIDRIRERAEDSGNESDGDQEELSALVEMGHHAPWDVNDE	WITO wild type
MQPLEILAVVALVVALILAIVVWTIVYIEYRKIQKQKKIDRLIDRIRERAEDSGNESDGD**AAA**LSALVEMGHHAPWDVNDE	61-63AAA
MQPLEILAVVALVVALILAIVVWTIVYIEYRKIQKQKKIDRLIDRIRERAEDSGNESDGDQEE**AAA**LVEMGHHAPWDVNDE	64-66AAA
MQPLEILAVVALVVALILAIVVWTIVYIEYRKIQKQKKIDRLIDRIRERAEDSGNESDGDQEELSA**AAA**MGHHAPWDVNDE	67-69AAA
MQPLEILAVVALVVALILAIVVWTIVYIEYRKIQKQKKIDRLIDRIRERAEDSGNESDGDQEELSALVE**AAA**HAPWDVNDE	70-72AAA
MQPLEILAVVALVVALILAIVVWTIVYIEYRKIQKQKKIDRLIDRIRERAEDSGNESDGDQEELSALVEMGH**AAA**WDVNDE	73-75AAA
MQPLEILAVVALVVALILAIVVWTIVYIEYRKIQKQKKIDRLIDRIRERAEDSGNESDGDQEELSALVEMGHHAP**AAA**NDE	76-78AAA
MQPLEILAVVALVVALILAIVVWTIVYIEYRKIQKQKKIDRLIDRIRERAEDSGNESDGDQEELSALVEMGHHAPWDV**AAA**	79-81AAA

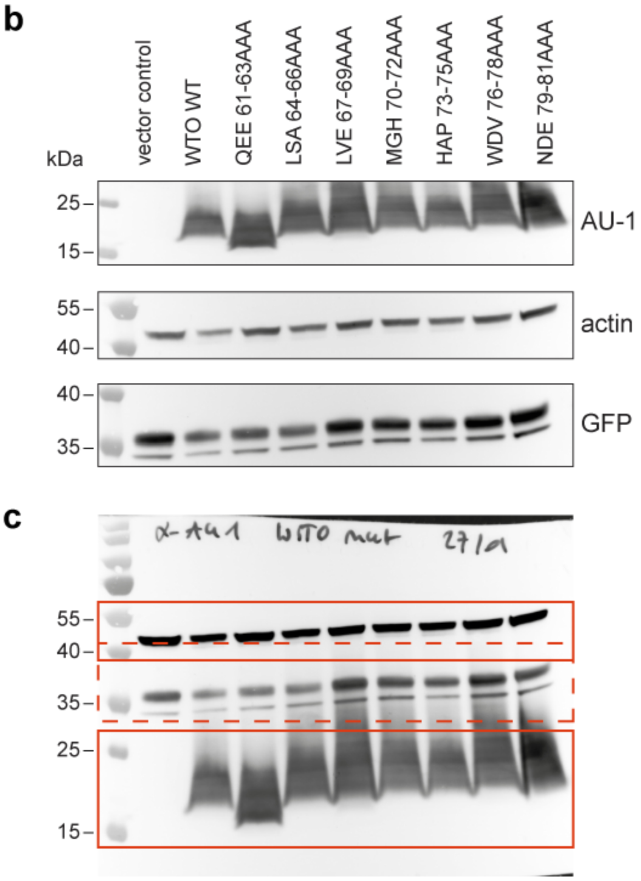

Supplementary Figure S5 Expression of WITO Vpu triple-alanine mutants.

(a) Schematic representation of WITO Vpu triple-alanine mutants; bold letters indicate amino acid exchanges. (b) 293T cells were transfected with pCG-IRES-eGFP plasmids encoding the indicated AU1-tagged Vpu proteins. At 24 h post transfection, cell lysates were prepared and analyzed by Western Blot using goat anti-AU1 and mouse anti-actin antibodies followed by HRP-conjugated anti-goat and anti-mouse IgG, respectively. HRP-conjugated anti-GFP staining served as transfection control. (c) Uncropped full-size blots with cropped regions indicated by red squares.

a

WITO (subtype B) and ZM247F (subtype C) Vpu mutants

MQPLEILAVVALVVALILAIVVWTIVYIEYRKIQKQKKIDRLIDRIRERAEDSGNESDGDQEELSALVEMGHHAPWDVNDE	WITO	wild type
MQPLEILAVVALVVALILAIVVWTIVYIEYRKIQKQKKIDRLIDRIRERAEDSGNESDGDQEELSALVEMGHH**RLL**DVNDE		APW/RLL
MQPLEILAVVALVVALILAIVVWTIVYIEYRKIQKQKKIDRLIDRIRERAEDSGNESDGDQEELSALVEMGHHAPW-----		Δ5
MQPLEILAVVALVVALILAIVVWTIVYIEYRKIQKQKKIDRLIDRIRERAEDSGNESDGDQEELSALVEMG----------		Δ10
MLELDYKIAIAALIVALIIAIVVWTIVYIEYRKLVRQRKIDWLIKRIRERAEDSGNESDGDQEELSTMVDMGHIRLLGAIDL	ZM247F	wild type
MLELDYKIAIAALIVALIIAIVVWTIVYIEYRKLVRQRKIDWLIKRIRERAEDSGNESDGDQEELSTMVDMGHI**APW**GAIDL		RLL/APW
MLELDYKIAIAALIVALIIAIVVWTIVYIEYRKLVRQRKIDWLIKRIRERAEDSGNESDGDQEELSTMVDMGHIRLL-----		Δ5
MLELDYKIAIAALIVALIIAIVVWTIVYIEYRKLVRQRKIDWLIKRIRERAEDSGNESDGDQEELSTMVDMG----------		Δ10

b

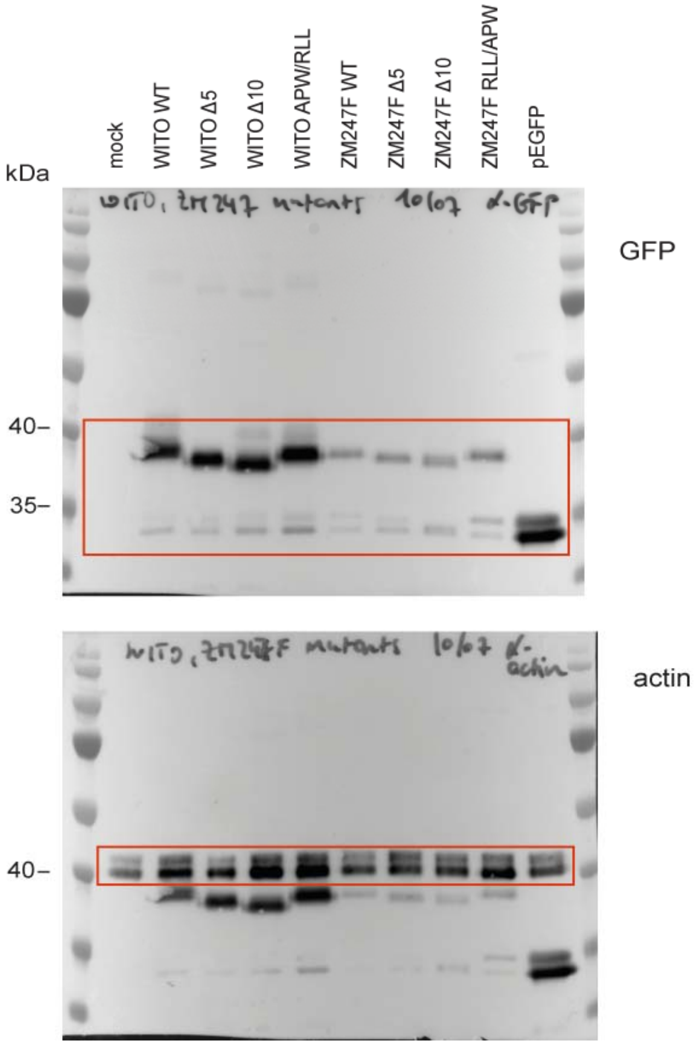

Supplementary Figure S6

(a) Schematic representation of WITO and ZM247F Vpu mutants; bold letters indicate amino acid exchanges, dashed indicate amino acid deletions. (b) Original uncropped full-size blots of Figure 5C; cropped regions are indicated by red squares.

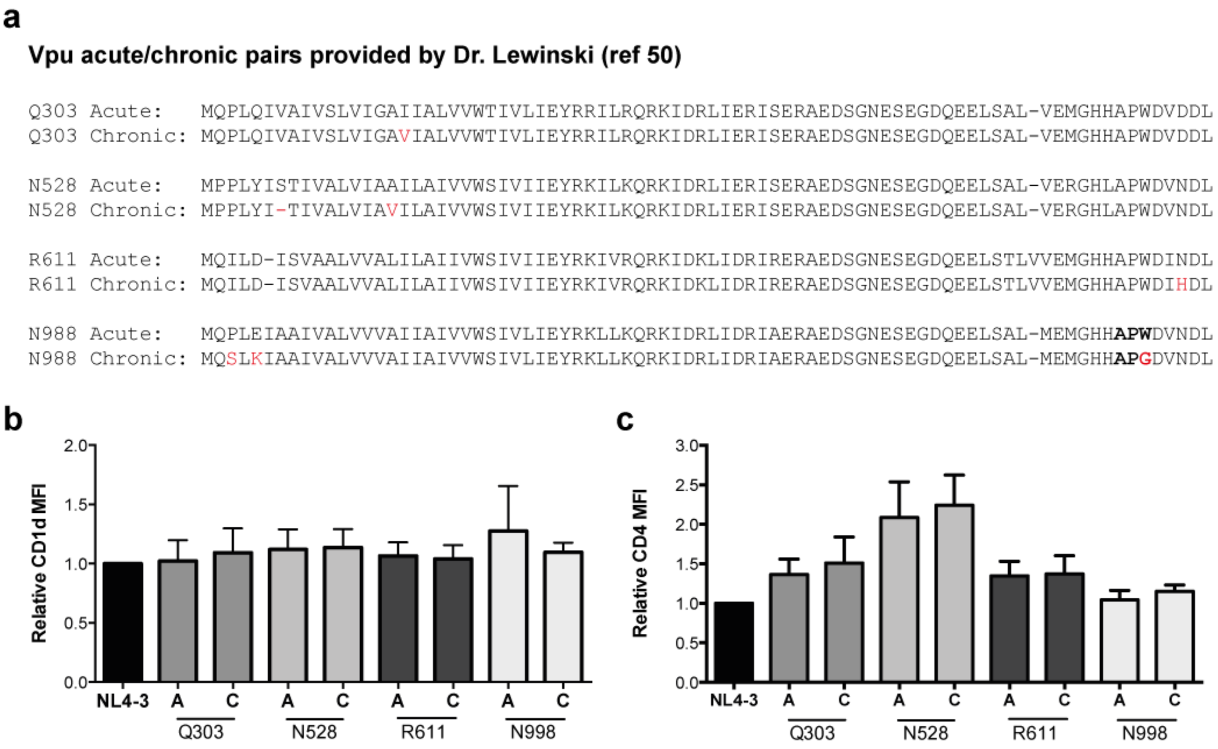

Supplementary Figure S7 CD1d down-regulation by Vpu protein pairs isolated during acute and chronic phases of infection.

(a) Amino acid sequences of acute and chronic Vpu protein pairs [49]. Differences between the pairs are highlighted in red; the APW motif is highlighted in bold. (b, c) 293T cells were co-transfected with CD1d or CD4 and the indicated Vpu protein pairs derived from viruses isolated during acute and chronic phases of infection. Data are expressed as surface MFIs relative to NL4-3 Vpu. Statistical analysis was done using one-way ANOVA with Sidak's multiple comparisons test. Average values from 3 independent experiments performed in duplicates (±SD) are shown.

Supplementary Table S1 Overview of HIV-1 and SIV *vpu* alleles analyzed in this study.

HIV-1 Clone	Group/Subtype	GenBank Accession
NL4-3	HIV-1 M/B	U26942
89.6	HIV-1 M/B	U39362
JR-CSF	HIV-1 M/B	M38429
YU-2	HIV-1 M/B	M89973
WITO.c	HIV-1 M/B	AY835451
RHPA.c	HIV-1 M/B	AY835447
THRO.c	HIV-1 M/B	AY835448
REJO.c	HIV-1 M/B	AY835449
CH040.c	HIV-1 M/B	GQ925946
CH058.c	HIV-1 M/B	GQ925948
CH077.t	HIV-1 M/B	GQ925949
CH106.c	HIV-1 M/B	GQ925947
KE.00	HIV-1 M/C	AF457054
ZM246F	HIV-1 M/C	FJ496185
ZM247F	HIV-1 M/C	FJ496195
CH198	HIV-1 M/C	KC156130
ZM249	HIV-1 M/C	DQ388514
CH167	HIV-1 M/C	KC156213
CH293	HIV-1 M/C	KC156216
CH432	HIV-1 M/C	KC156218
CH534	HIV-1 M/C	KC156221
SE.95	HIV-1 M/A1	AF069673
CY.94	HIV-1 M/A2	AF286237
UG.99	HIV-1 M/D	AF484498
ZA.90	HIV-1 M/D	EF633445
BE.93	HIV-1 M/F1	AF077336
CM.02	HIV-1 M/F2	AY371158
PT.x	HIV-1 M/G	AY612637
GH.03	HIV-1 M/G	AB287004
BE.93	HIV-1 M/H	AF190128
SE.94	HIV-1 M/J	AF082395
CD.97	HIV-1 M/K	AJ249235
MVP13127	HIV-1 O	AF316856
9435	HIV-1 O	GQ925939
HJ001	HIV-1 O	GQ925937
HJ100	HIV-1 O	GQ925943
HJ162	HIV-1 O	GQ925941
HJ256	HIV-1 O	GQ925940
HJ389	HIV-1 O	GQ925945
HJ428	HIV-1 O	GQ925942
HJ736	HIV-1 O	GQ925944
HJ036	HIV-1 O	GQ925938
YBF30	HIV-1 N	AJ564926
2693BA	HIV-1 N	GQ925950
CK1.62	HIV-1 N	GQ925951
06CM-U14842	HIV-1 N	GQ324958
06CM-U14296	HIV-1 N	GQ324962
02CM-SJGddd	HIV-1 N	GQ324959
RBF168	HIV-1 P	GQ328744
U14788	HIV-1 P	HQ179987
**SIV Clone**	**Species *Subspecies***	**GenBank Accession**
GAB1	SIVcpz*Ptt* (Chimpanzee, *Pan troglodytes troglodytes*)	X52154
MB66	SIVcpz*Ptt*	DQ373063
MT145	SIVcpz*Ptt*	DQ373066
LB7	SIVcpz*Ptt*	DQ373064
EK505	SIVcpz*Ptt*	DQ373065
MB897	SIVcpz*Ptt*	EF535994
CP2139	SIVgor (Gorilla, *Gorilla gorilla gorilla*)	FJ424866
gsn71	SIVgsn (Greater spot-nosed monkey, *Cercopithecus nictitans*)	AF468658
gsn166	SIVgsn	AF468659
monCML1	SIVmon (Mona monkey, *Cercopithecus mona*)	AY340701
mus1085	SIVmus (Mustached monkey, *Cercopithecus cephus*)	AY340700
muss1239	SIVmus	EF070330
muss2500	SIVmus	EF070331
